# RNA-seq data science: From raw data to effective interpretation

**DOI:** 10.3389/fgene.2023.997383

**Published:** 2023-03-13

**Authors:** Dhrithi Deshpande, Karishma Chhugani, Yutong Chang, Aaron Karlsberg, Caitlin Loeffler, Jinyang Zhang, Agata Muszyńska, Viorel Munteanu, Harry Yang, Jeremy Rotman, Laura Tao, Brunilda Balliu, Elizabeth Tseng, Eleazar Eskin, Fangqing Zhao, Pejman Mohammadi, Paweł P. Łabaj, Serghei Mangul

**Affiliations:** ^1^ Department of Pharmacology and Pharmaceutical Sciences, USC Alfred E. Mann School of Pharmacy and Pharmaceutical Sciences, Los Angeles, CA, United States; ^2^ Department of Clinical Pharmacy, USC Alfred E. Mann School of Pharmacy and Pharmaceutical Sciences, Los Angeles, CA, United States; ^3^ Department of Computer Science, University of California, Los Angeles, CA, United States; ^4^ Beijing Institutes of Life Science, Chinese Academy of Sciences, Beijing, China; ^5^ Małopolska Centre of Biotechnology, Jagiellonian University, Krakow, Poland; ^6^ Institute of Automatic Control, Electronics and Computer Science, Silesian University of Technology, Gliwice, Poland; ^7^ Department of Computers, Informatics and Microelectronics, Technical University of Moldova, Chisinau, Moldova; ^8^ Department of Microbiology, Immunology and Molecular Genetics, University of California Los Angeles, Los Angeles, CA, United States; ^9^ Department of Computational Medicine, David Geffen School of Medicine at UCLA, CHS, Los Angeles, CA, United States; ^10^ Pacific Biosciences, Menlo Park, CA, United States; ^11^ Department of Human Genetics, David Geffen School of Medicine at UCLA, Los Angeles, CA, United States; ^12^ Key Laboratory of Systems Biology, Hangzhou Institute for Advanced Study, University of Chinese Academy of Sciences, Hangzhou, China; ^13^ Department of Integrative Structural and Computational Biology, The Scripps Research Institute, La Jolla, CA, United States; ^14^ Department of Biotechnology, Boku University Vienna, Vienna, Austria; ^15^ Department of Quantitative and Computational Biology, USC Dornsife College of Letters, Arts and Sciences, Los Angeles, CA, United States

**Keywords:** RNA sequencing, transcriptome quantification, differential gene expression, high throughput sequencing, read alignment, bioinformatics

## Abstract

RNA sequencing (RNA-seq) has become an exemplary technology in modern biology and clinical science. Its immense popularity is due in large part to the continuous efforts of the bioinformatics community to develop accurate and scalable computational tools to analyze the enormous amounts of transcriptomic data that it produces. RNA-seq analysis enables genes and their corresponding *transcripts* to be probed for a variety of purposes, such as detecting novel exons or whole transcripts, assessing expression of genes and alternative transcripts, and studying alternative splicing structure. It can be a challenge, however, to obtain meaningful biological signals from raw RNA-seq data because of the enormous scale of the data as well as the inherent limitations of different sequencing technologies, such as *amplification bias* or *biases of library preparation*. The need to overcome these technical challenges has pushed the rapid development of novel computational tools, which have evolved and diversified in accordance with technological advancements, leading to the current myriad of RNA-seq tools. These tools, combined with the diverse computational skill sets of biomedical researchers, help to unlock the full potential of RNA-seq. The purpose of this review is to explain basic concepts in the computational analysis of RNA-seq data and define discipline-specific jargon.

## 1 Introduction

High-throughput DNA sequencing technologies, including *next-generation sequencing* and the newly emerging *third-generation sequencing*, enable the gene sequences of living organisms to be probed in a cost-effective manner (Shendure and Ji, 2008). These sequencing technologies have also been adapted for RNA sequencing (RNA-seq), which enables the expression of various RNA populations, including mRNA and total RNA, to be detected and quantified. RNA-seq has reshaped biomedical research by expanding researchers’ ability to analyze a vast range of biological data (Kukurba and Montgomery, 2015). To derive biological insights from RNA-seq data, researchers need to understand the steps involved in RNA-seq analysis and select appropriate tools to answer their research question.

Biomedical researchers are often tasked with using computational methods for RNA-seq analysis, which are typically available wrapped as software tools and packages. In this review, we provide an overview of diverse methodologies for RNA-seq analyses that can be used to detect novel exons and transcripts, quantify gene expression and alternative splicing, and study alternative splicing structure. We discuss the steps from the generation of raw data using sequencing technologies to the effective interpretation and visualization of RNA-seq data using mapping and quantification techniques. By summarizing the biological and computational foundations of RNA-seq data generation, analysis, and software development, we hope this review will lead to a more deliberate use of existing computational tools.

## 2 RNA sequencing

RNA-seq uses high-throughput sequencing of nucleic acids to determine the nucleotide sequence of RNA molecules as well as the quantities of specific RNA species within populations of RNA molecules. RNA-seq analysis requires specialized computational tools that can account for the shortcomings of sequencing technologies, including the generation of *sequencing errors* (Le et al., 2013), *length biases* (Oshlack and Wakefield, 2009), and *fragmentation* (Tuerk et al., 2017). Computational analysis of RNA-seq data has led to many scientific advances, including novel therapeutic discoveries, detailed understanding of genetic regulatory regions, and identification of biomarkers and pathogenic mutations (Han et al., 2015).

**FIGURE 1:**
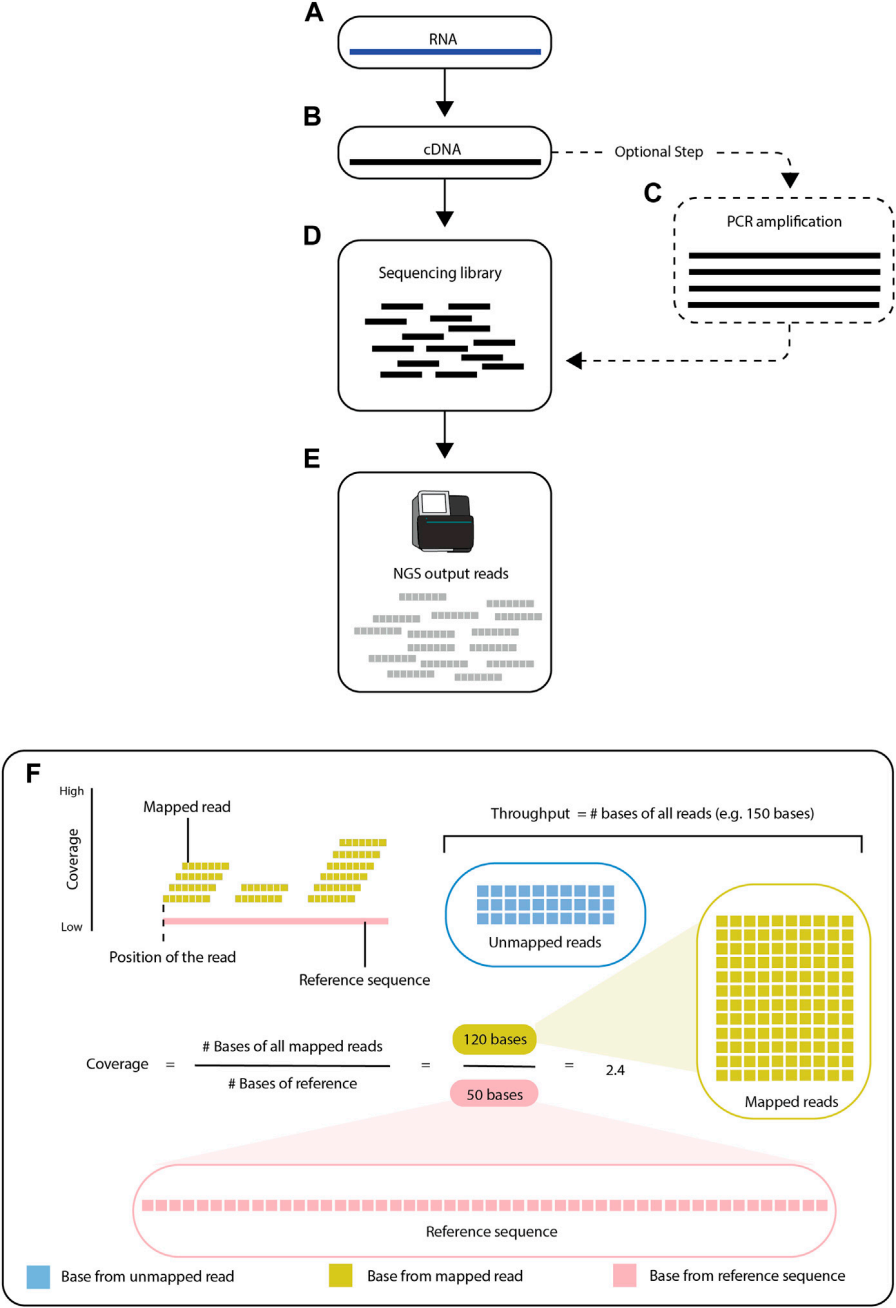
Overview of RNA-seq. RNA-seq is a process of creating short sequencing reads from RNA molecules. The steps consist of first converting the RNA **(A)** into cDNA **(B)**, then (optionally) amplifying the cDNA by PCR **(C)**, and finally fragmenting the cDNA into short pieces (known as fragments). After the sequencing library **(D)** is prepared, the fragments are used as input for next-generation sequencing **(E)**. The resulting sequence reads contained in FASTQ files are then aligned to a reference sequence **(F)**. Modern high-throughput sequencing machines can generate up to 150 million reads per run. The reference sequence, shown as a pink line, is known. The goal of the alignment is to find the *locus* in the reference sequence with the greatest match to each read. Reads are shown to align to the specific positions/locations and these mapped locations are recorded.

Preparation of an RNA-seq library starts with extraction and isolation of RNA from a biological sample, such as a cell line or a frozen tissue sample. For RNA-seq performed with short-read sequencing (see Section 2.1), the isolated RNA is reverse-transcribed and converted into *cDNA*, which is then amplified by *polymerase chain reaction (PCR)* and fragmented into short sequences (either before or after PCR) (Prakash and Haeseler, 2017) (Figure 1). After the RNA molecules are processed, the RNA-seq library becomes the input for a sequencing platform (Kukurba and Montgomery, 2015), which generates reads (i.e., the sequenced fragments from the RNA-seq library).

### 2.1 High-throughput RNA-seq technologies

High-throughput sequencing techniques can derive millions of nucleotide sequences from an individual *transcriptome* (Stark et al., 2019). These nucleotide sequences provide multifold coverage of the whole transcriptome. High-resolution RNA-seq can identify which genes are actively transcribed in a sample and quantify the levels at which alternative transcripts of a gene are transcribed (Gerstein et al., 2007). The reads generated by different sequencing technologies have lengths ranging from hundreds of base pairs (usually referred to as short reads) to thousands of base pairs (referred to as long reads) (Shendure and Ji, 2008; Haas and Zody, 2010; Pollard et al., 2018). Illumina, Nanopore, and PacBio are among the most commonly used high-throughput sequencing platforms (Ye et al., 2015).

Illumina sequencing, considered a next-generation sequencing technology, is based on sequencing-by-synthesis chemistry and was first commercialized in 2006 (Shen and Shen, 2019). For Illumina RNA-seq, isolated RNAs are reverse-transcribed into single-stranded cDNA, which is then ligated to synthetic adapters, immobilized on a solid surface, and amplified by PCR. Then, a reaction mixture is added containing primers, DNA polymerase, and modified nucleotides. The modified *nucleotides have a fluorescent label* that serves as both a reversible terminator of DNA synthesis and an indicator of which nitrogenous base the nucleotide contains. As a new strand of DNA is synthesized using the immobilized cDNA as a template, each incorporated nucleotide is detected with a *charge-coupled device (CCD)* camera and identified by the color of the fluorescent label. The fluorescent label is then removed, and the next nucleotide is added in a new round of DNA synthesis. This cycle is repeated until each base in the cDNA is identified. The sequences of more than 10 million cDNA fragments can be simultaneously determined in parallel using the Illumina platform, giving rise to higher sequencing throughput compared with other sequencing platforms (Morganti et al., 2019; Workflows for RNA Sequencing, 2023).

Nanopore sequencing, which serves as the basis for the MinION, GridIOn, and PromethION platforms, was first introduced in 2014 by Oxford Nanopore Technologies. Nanopore sequencing can produce short or long reads from native DNA and RNA fragments of any length. Nanopores are very small holes in a membrane that can be created by pore-forming proteins or by non-biological means. The Nanopore sequencing method simultaneously sends an ionic current and a single strand of DNA or RNA through a nanopore. As the ionic current passes through each nucleotide that successively occupies the nanopore, it undergoes disruptions that are unique to the nitrogenous base. The patterns of disruption in the current can be interpreted to identify each base in the DNA or RNA strand that passes through the nanopore. Whereas short-read sequencing technologies such as Illumina require chemical modification or PCR amplification, Nanopore technology is capable of sequencing DNA or RNA without these additional steps, making it a third-generation sequencing technology (Bharagava et al., 2019).

PacBio sequencing, also known as SMRT (single-molecule, real-time) sequencing, was introduced in 2010 and generates full-length cDNA sequences (i.e., long reads) that characterize transcripts of targeted genes or across entire transcriptomes. Long reads generated by PacBio are accurate at the scale of a single molecule because they are generated by a process of circular consensus sequencing, in which the same cDNA is effectively read many times (Eid et al., 2009; Vierra et al., 2021). The comparatively high sensitivity of PacBio can be limited by external factors. For example, PacBio can produce full-length cDNA during the library preparation step; however, it can only generate high-quality reads if the target cDNA is short enough to be sequenced in multiple passes.

**FIGURE 2:**
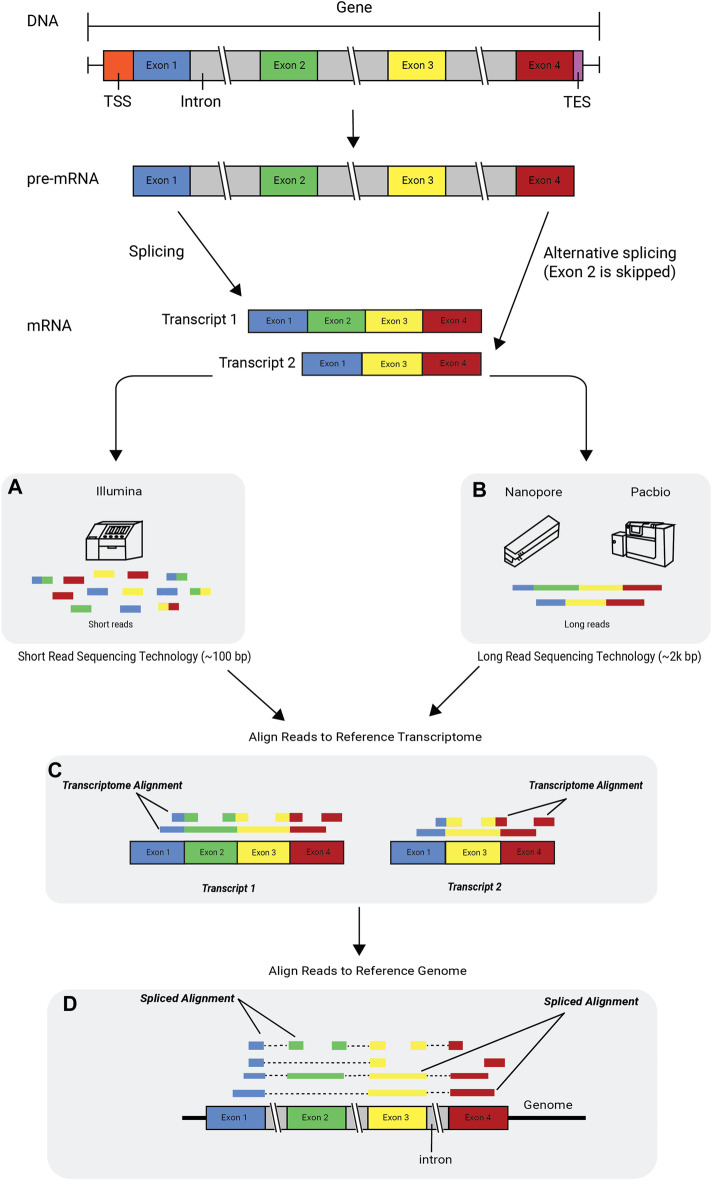
Alternative splicing and RNA-seq technologies. The flow of genetic information begins with DNA, which consists of introns and exons. DNA is transcribed into pre-mRNA and then further processed into mature mRNA by splicing out the introns and leaving the exons glued together. The mRNA is then translated into a protein. Transcripts with different arrangements of exons can be formed in a process called alternative splicing or exon skipping. An RNA-seq read is a short sequence sampled from a transcript. Reads are generated using sequencing technologies such as **(A)** the Illumina platform, which produces short reads, and the **(B)** Nanopore and PacBio platforms, which produce long reads. The figure depicts two scenarios in which uniquely mapped reads are aligned to a reference transcriptome **(C)** and a reference genome **(D)**, respectively. A few of the reads are multicolored, indicating that when aligned, they span across an exon-exon junction. Some of the shorter reads (single-colored) are aligned only to a single exon and do not span across the junction. *TSS*, transcription start site; *TES*, transcription end site.

Each sequencing technology has inherent advantages and limitations, so no technology is best suited for all types of RNA-seq analysis (Box 1). Short-read technologies can generate data with a lower error rate and higher throughput than long-read technologies; however, the short-read length makes reconstruction and quantification of the transcriptome challenging (Korf, 2013; The RGASP Consortium et al., 2013a; The RGASP Consortium et al., 2013b; Amarasinghe et al., 2020). Long-read sequencing improves the accuracy of assembly (concatenation of individual reads to reassemble the transcriptome), or can even eliminate the need for assembly, as each read can cover an entire transcript. Long-read sequencing can also be used to produce complete, unambiguous information about alternative splicing, gene structure, regulatory elements, and coding regions. Long-read sequencing currently has a higher error rate and lower throughput compared with short-read sequencing, however (Figure 2) (Sedlazeck et al., 2018; De Maio et al., 2019; Mahmoud et al., 2019). Hybrid approaches that combine long reads and short reads can eliminate the limitations of each separate approach and can be used to accurately quantify and assemble known and novel transcripts (De Maio et al., 2019; Amarasinghe et al., 2020; Berbers et al., 2020), but they also have higher costs and more material requirements. Data gathered using Illumina, Nanopore, and PacBio sequencing technologies can be used to address a wide range of research areas, including transcriptome analysis, population-scale analysis, and clinical research (Wang et al., 2021).

Box 1 **| Advantages and limitations of short and long reads**

i. **Error rate**—Short read sequencing technologies have a lower error rate when compared to long read sequencing technologies **(a, b)**.

ii. **Throughput**—The throughput of long read sequencing technologies is typically lower than the throughput of short read sequencing technologies **(c)**.

iii. **Alignment**—Short reads suffer from multi-mapping issues, whereas longer reads, by nature of having more information, can be more accurately mapped to its origin. Due to a high error rate, pairwise alignment between the read, the reference transcriptome, and/or genome is more challenging for long reads compared to short reads.

iv. **Assemble novel transcripts**—Longer reads are preferred for *de novo* assembly, because they make the assembly step efficient. Most short reads do not span the shared region or shared exon junction, making the assembly step ambiguous. Full-length transcript sequencing eliminates the need for assembly.

v. **Estimate transcripts and gene expression**—Shorter reads are preferred for quantification of transcripts due to their higher throughput. However, assigning short reads to the transcripts requires more advanced probabilistic and statistical approaches. Longer reads have lower throughput, but they can usually cover the entire transcript and make determination of the transcript for each read a straightforward process.

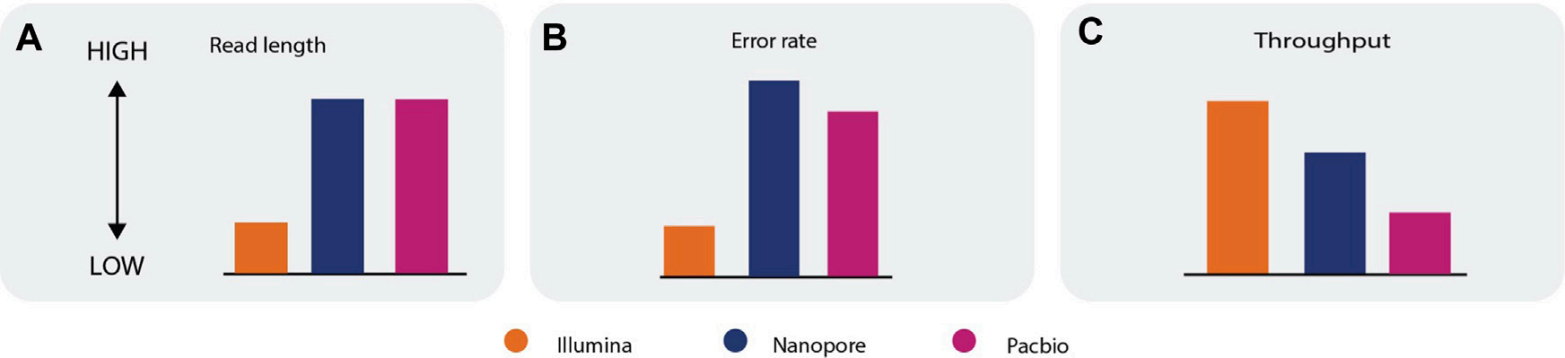

## 3 RNA-seq data science: From raw data to effective interpretation

RNA-seq is multifaceted and can be used to uncover and expound new insights on, for example, a dysregulated gene or defective protein that has a downstream effect leading to a disease state (Costa et al., 2010). Computational analysis of RNA-seq data is central to decoding the biological complexities in the transcriptomes of living organisms, including humans (Costa et al., 2010). Here, we describe the major steps of computational analysis of RNA-seq data, beginning from the processing of raw data to the uncovering of biological insights.

### 3.1 Quality control of raw data

During the sequencing process, errors are introduced into reads that can bias the results of downstream analyses. Read trimming and data quality control to filter and assess the quality of raw reads (Yang et al., 2013) are therefore essential after the reads have been generated. Read trimming removes adapter sequences and portions of reads with low accuracy, as indicated by a low *PHRED quality score* (Martin, 2011; Dodt et al., 2012; Bolger et al., 2014). In addition, computational error correction can be applied to reduce the number of sequencing errors (Lima et al., 2020; Mitchell et al., 2020).

## 4 Read alignment

Read alignment is an essential step in RNA-seq downstream analysis. RNA-seq data typically lack information about the order and origin of the reads, including the specific part, homolog, or strand of the *genome* from which they originate. Computational alignment of the reads to an annotated reference transcriptome can establish where on the genome the reads originated (Figure 1) (Brown, 2002). Alignment of the reads to a reference sequence also reveals how many reads overlap each position on the reference sequence, which is known as the coverage. There are several bioinformatics tools (e.g., GenomeScope (Vurture et al., 2017), Smudgeplot (Ranallo-Benavidez et al., 2020), and Merqury (Rhie et al., 2020)) that can estimate the coverage without mapping the reads to a reference sequence (Ranallo-Benavidez et al., 2020; Rhie et al., 2020), as most of the overlap between reads is preserved with or without the reference sequence (Vurture et al., 2017) (Figure 1).

Alignment of RNA-seq reads to a complementary reference sequence can help determine which transcripts are expressed and the degree to which they are expressed, but the alignment approach is ill-equipped to discover transcripts that are missing from the reference sequence. Furthermore, even the human reference transcriptome remains incomplete (Nellore et al., 2016). Novel transcripts can be discovered by performing *de novo* assembly of RNA-seq reads to generate an entire transcriptome without alignment to a reference sequence; however, this can be challenging and requires large amounts of computational time and resources (Grabherr et al., 2011) As an alternative, RNA-seq reads can be aligned to curated databases of known transcripts such as RefSeq (Pruitt et al., 2007), UCSC genome browser, Ensembl, GENCODE (GENCODE, 2022), and AceView (Larsson et al., 2005), and reads that fail to align to known transcripts can then be aligned to a reference genome to identify novel transcripts.

One computational challenge in aligning RNA-seq reads to a reference genome is the handling of spliced junctions, where one part of the read maps to the end of one exon and the rest of the read maps to another exon, which may be located thousands of base pairs away from the first exon. Spliced junctions are the result of the removal of non-coding parts of a gene, called introns, and the splicing together of the coding parts of the gene, called exons. Genes can generate multiple mRNA transcripts through alternative splicing. As a result, exons are combined or skipped in different ways and have alternative start/end sites. These varying combinations create different transcripts, known as *isoforms*, from the same gene. As a biological process, alternative splicing is evolutionarily advantageous, because it enables the production of different protein variants from the same genetic information (Figure 2). When genome annotations are available, existing exon structures can be used to map reads across known splice junctions; however, this knowledge-guided approach may be biased towards mapping only known junctions while failing to discover novel ones.

In cases where reads align to multiple transcripts, it might not be possible to discern from which transcript the reads originate. Splice alignment software packages (Wang et al., 2010; Dobin et al., 2013; Kim et al., 2019) are designed to minimize multi-mapping by correctly aligning reads across the exon–intron junctions of the reference genome (Figure 2). This can be a crucial first step of reference-guided assembly, wherein transcripts that are present in the sample but not annotated in the reference are assembled using the spliced read alignments to the reference.

In some instances, reads do not perfectly align with the reference sequence but instead contain mismatches, which can be caused either by sequencing errors or by biological variation such as mutations (Mitchell et al., 2020). RNA-seq alignment tools are typically equipped with a customizable threshold for tolerating mismatches in the alignment; however, it is important to distinguish between sequencing errors and real variation between the transcripts and the reference sequence. Specialized computational tools (Abate et al., 2014; Fernandez-Cuesta et al., 2015) can identify and classify genes using strategies such as *de novo* assembly (assembly of reads without alignment to a reference sequence), identification of reads that span fusion junctions, and filtering of gene fusion candidates based on various criteria.

## 5 Quantitative analysis of gene expression

RNA-seq enables quantitative analysis of gene expression at the level of alternative transcripts. The sequence fragments derived from mRNA can reveal which genes are expressed and how strongly they are expressed. Additionally, differential expression (DE) analysis can show how expression levels change under different conditions or between different populations.

### 5.1 Estimation of transcript and gene expression

Computational methods can estimate expression levels of genes and transcripts by counting the number of reads that match individual reference transcripts. Tools like HT-Seq-count, Rcount, and featureCounts (Liao et al., 2014; Anders et al., 2015; Schmid and Grossniklaus, 2015) are highly robust and widely used for such analyses; however, counting-based tools are ill-equipped to estimate the expression levels of different isoforms of expressed genes using short reads, as the majority of isoforms share a large percentage of exons and cannot be uniquely assigned to individual transcripts (Figure 2). The shorter the reads, the greater the probability that they will match multiple transcripts. A conservative approach to tackle this challenge is to consider only the reads that uniquely map to a single transcript (e.g., reads that map to transcript-specific splicing junctions or exons) (Conesa et al., 2016). An alternative approach that utilizes a larger fraction of the RNA-seq reads is to probabilistically assign reads to the isoforms from which they likely originated (Li and Dewey, 2011; Nicolae et al., 2011; Trapnell et al., 2012; Pertea et al., 2015).

A number of approaches quantify gene expression using complete read alignment, which requires large amounts of computational power and time to compare each read to reference sequences base-by-base. Pseudoalignment methods have been developed as an alternative approach that has a much smaller computational burden. These methods forgo the base-by-base accuracy of alignment and determine an approximate alignment of the reads on the genome, which is still sufficiently accurate to quantify gene expression. Pseudoalignment algorithms leverage a pre-compiled library of unique k-mers (exact substrings of length k) contained in known transcripts and assign reads to transcripts by counting the k-mer occurrences in the reads, thus achieving up to 100 times faster quantification compared with alignment-based methods (Bray et al., 2016). Sailfish (the pioneer of pseudoalignment) (Patro et al., 2014), Salmon (Patro et al., 2017), and Kallisto (Bray et al., 2016) each utilize pseudo-alignment-based algorithms to quantify the isoforms of expressed transcripts (Alser et al., 2020), each providing comparable accuracy in expression quantification. A more detailed explanation of these tools can be found in Supplementary Material S2.

### 5.2 Differential gene expression analysis

After gene and transcript expression levels are estimated, statistical approaches are employed to detect differences in expression levels across experimental groups (e.g., different sexes or cohorts exposed to different environmental conditions) (Conesa et al., 2016). Expression levels measured for the same gene under different conditions cannot be directly compared, as each experiment represents a statistical sample, giving only the relative mRNA levels in comparison to the other mRNAs present in the sample. In addition, mRNA levels change over time, and reads can align to multiple places, making exact quantitation difficult. The purpose of statistical testing is to ensure that an observed change in mRNA levels is due to an actual difference in expression between experimental conditions.

To test whether the expression of a given gene is different between two groups, measurements are repeated in multiple replicates of the same experiments, and then a statistical test is applied. Through this process, the variation in expression between different conditions can be compared to the variation within replicates of the same condition. Each statistical test is based on a null hypothesis that the gene expression is the same between groups, which is usually true for the majority of genes. The value that indicates whether there is likely to be a true difference between groups is called the *p*-value, which gives the probability of observing a particular difference, or a more extreme difference, assuming that the null hypothesis is true. Small *p*-values give strong evidence against the null hypothesis. Genes with low *p*-values are considered to be differentially expressed, and the null hypothesis is rejected for those genes. The typical threshold for rejection of a null hypothesis is a *p*-value less than 0.05, but this cutoff is arbitrary and might need to be altered depending on how noisy the data are (Liu et al., 2006; Glaus et al., 2012; Shastry et al., 2020).

There are two types of error associated with statistical tests: Type I error and Type II error. A Type I error occurs if a test rejects a true null hypothesis. A Type II error occurs if a test accepts a false null hypothesis. The *p*-value indicates the probability of making a Type I error in a given test. For example, if the *p*-value threshold is set at 0.05 (i.e., 5%), and 20,000 genes are being tested, then 1,000 genes (5% ⋅ 20,000) will be wrongly considered to be differentially expressed because of Type I errors. There are two approaches to control Type I errors, also referred to as false positives. One approach is to control the family-wise error or the probability that there is at least one Type I error among all the rejected null hypotheses. The other approach is to control the false discovery rate, or the proportion of Type I errors among all the rejected null hypotheses. Both approaches involve calculation of an adjusted *p*-value (p-adj) for each gene, which can then be used for further analysis (Jafari and Ansari-Pour, 2018).

It is important to account for *noise* which includes sources of variation that are unrelated to the experimental variable of interest, when performing differential expression analysis. For example, *batch effects*, or confounding factors arising from samples being tested on different days, by different laboratory technicians, or in different laboratories (technical batch effects), can result in unwanted differences in measured values. In addition, variation due to intrinsic factors such as high GC content or gene body coverage evenness (biological batch effects) can affect the quantification of technical replicates of a sample. Existing statistical methods can effectively detect and adjust for hidden confounding factors (Li et al., 2014).

Other approaches to differential expression analysis that can produce more accurate results than conventional p-adj values use different metrics such as the minimum significant difference or the generalized linear model (GLM) framework (McCarthy et al., 2012), where a combination of *p*-values and log fold changes is applied to identify the genes or transcripts with the most significant differences in expression. Another alternative approach is the probability of positive log ratio (PPLR) (Liu et al., 2006), which was initially developed for microarray analysis and subsequently adjusted for RNA-seq data (Glaus et al., 2012). The PPLR uses a Bayesian hierarchical model to express the probability that the ratio of expression levels between two conditions is positive (i.e., the expression is upregulated in the second condition relative to the first). A PPLR value close to 1 means there is a very high probability that a given transcript is upregulated in the second condition (Liu et al., 2006). When the PPLR value is close to 0, there is a very low probability of upregulation, and consequently a high probability of downregulation, in the second condition relative to the first. There is no direct relation between PPLRs and *p*-values, as they look at the problem from different perspectives (i.e., in the probabilistic approach an uncertainty propagation between successive stages of analysis is possible and desired). Both approaches are capable of identifying large numbers of differentially expressed genomic features. If the number of differentially expressed features is too large, a more stringent cutoff for statistical significance can be applied to make the analysis more manageable.

Depending on the type of *normalization* performed on RNA-seq data, machine-learning approaches can be used to identify differentially expressed genes with classification models based on discrete or continuous distributions. Machine learning approaches have been used to manage, model, and categorize biological data, enabling high-impact discoveries in the field of biomedicine (Shastry et al., 2020). RNA-seq data are discrete in nature. The two most common ways to normalize RNA-seq data for machine learning-based differential expression analysis are to model the data as a Poisson or negative binomial distribution or transform the data to be similar to a distribution of microarray data. The Bioconductor MLSeq (Goksuluk et al., 2019) package is a comprehensive source of combinations of different normalization and machine-learning methods for RNA-seq analysis. After the data are normalized, genes or alternative transcripts (features) can be ranked, or standard sample classification can be performed, and the features that make the strongest contributions to the assignment of samples to particular groups can be extracted (Goksuluk et al., 2019). With a deep learning approach, it is also possible to predict differences in gene expression from histone modification signals (Sekhon et al., 2018).

Differential expression analysis can be complemented by *expression quantitative trait loci (eQTL)* analysis, which formally compares the expression levels of a given gene between groups with different copy numbers (0, 1, or 2) of the minor allele. Each read alignment technique produces different results, which may impact which genes are identified as differentially expressed (Castel et al., 2015). The power to detect differentially expressed genes and eQTLs depends on the sequencing depth of the sample, the minor allele frequency of the gene being tested, the expression level of the gene, and the length of the gene (McKenna et al., 2010). The magnitude of the eQTL can be quantified by the log allelic fold change (Hu et al., 2015), and its significance is tested using a binomial distribution or over-dispersed generalizations (Kumasaka et al., 2016; Knowles et al., 2017; Mohammadi et al., 2019; Zou et al., 2019; Wang et al., 2020). Some of the popular approaches to detect eQTLs use transformation and linear regression models (Shabalin, 2012; Ongen et al., 2016; Taylor-Weiner et al., 2019).

The results of differential expression analyses can be validated using independent techniques such as *quantitative PCR (qPCR)*, which is statistically assessable (Skelly et al., 2011). Measurements of gene expression obtained by qPCR are relatively similar to measurements obtained by RNA-seq analysis, where a value can be calculated for the concentration of a target region in a given sample (Harvey et al., 2015; Romanel et al., 2015; Xie et al., 2019). Additional information about quantification of RNA splicing and splicing QTL (sQTL) analyses can be found in Supplementary Material S3.

## 6 Measurement of allele-specific expression

**FIGURE 3:**
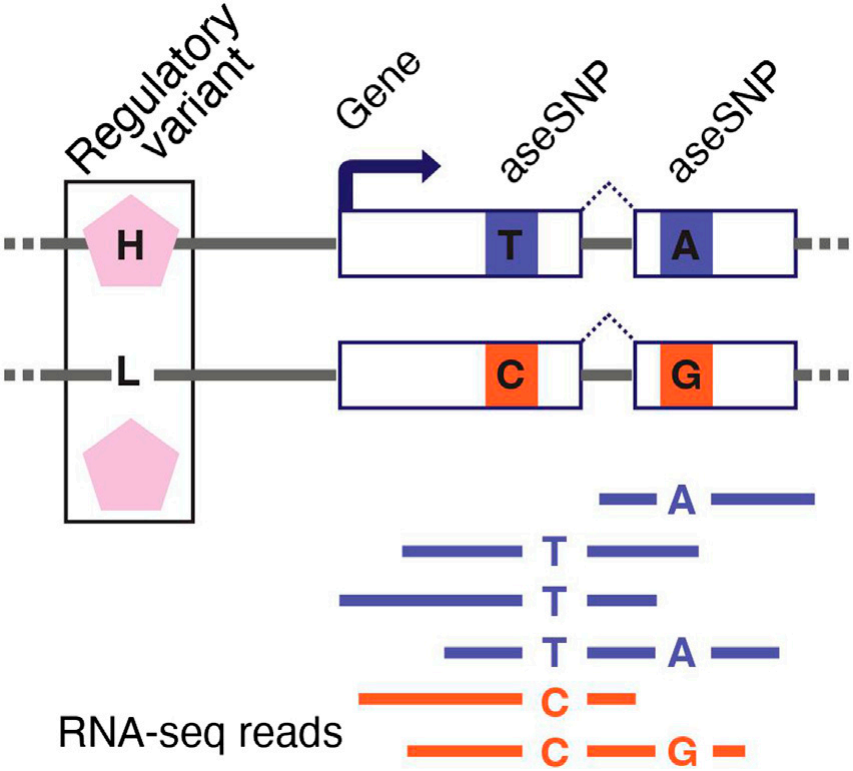
Measuring allele-specific expression with RNA-seq. RNA-seq can be used to generate allele-specific expression (ASE) data for genes with a heterozygous single-nucleotide polymorphism in the transcribed region (aseSNP). The aseSNP enables sequencing reads to be mapped to the haplotype from which they originate. Imbalance in ASE data is a functional indicator of a cis-regulatory difference between the two haplotypes that is driven by heterozygous regulatory variants. Data from multiple aseSNPs can be aggregated to improve ASE data quality. The non-coding regulatory variant depicted here has two alleles inducing higher (H) and lower (L) expression of the target gene.

RNA-seq can measure allele-specific expression (ASE or allelic expression) to uncover the cis-regulatory effects of genetic variants (McKenna et al., 2010; Castel et al., 2015; Raghupathy et al., 2018). ASE represents gene expression measured independently for the paternal and maternal alleles of a gene. In a typical RNA-seq experiment, ASE can be measured only in genes that contain a heterozygous *single-nucleotide polymorphism (SNP)* within the transcribed region. This SNP, referred to as the aseSNP, can be used as a tag to identify reads that originate from each copy of the gene (Figure 3).

Allelic imbalance—the ratio between paternal and maternal allele expression—identifies genetic cis-regulatory differences between two haplotypes. The log allelic fold change can also be calculated to quantify the magnitude of allelic imbalance (Hu et al., 2015). An aseSNP is not itself a regulatory variant and should not induce an imbalanced ASE signal. However, there can be a bias in ASE data that falsely suggests that the haplotype carrying the reference allele for the aseSNP has slightly higher expression across all genes. This issue, known as allelic bias or reference bias, can be mitigated in two ways: by aligning the RNA-seq reads to a personalized reference genome that excludes likely biased sites (Dobin et al., 2013; van de Geijn et al., 2015; Gao and Zhao, 2018; Kristensen et al., 2019; Ferraro et al., 2020), or by aggregating the ASE signal from multiple aseSNPs in each gene (Chen et al., 2021). ASE data can also be used to improve statistical power for identifying eQTLs (Gao and Zhao, 2018; Kristensen et al., 2019; Zou et al., 2019; Ferraro et al., 2020) and to map the causal regulatory variants in eQTL data (Kim and Salzberg, 2011; Gao et al., 2018; Haas et al., 2019). Furthermore, ASE data are inherently robust to noise, so they are useful for identifying gene-by-environment interaction effects (Li, 2013) or the effects of rare genetic variants on gene expression to improve diagnostic accuracy for Mendelian diseases (Hoffmann et al., 2014; Ji et al., 2019).

## 7 Profiling circular RNA with RNA-seq

Circular RNA (circRNA) is a large class of RNA molecules with a covalently closed circular structure that plays important roles in various biological processes and metabolic mechanisms (Wu et al., 2020). In recent years, a variety of computational tools have been developed for circRNA study (Gao and Zhao, 2018; Chen et al., 2021). Identification of circRNAs is based on detection of reads spanning the circle junction, termed the back-splice junction (BSJ). Most tools (Cheng et al., 2016; Zhang et al., 2016; Gao et al., 2018) employ aligners (Humphreys et al., 2019; Wu et al., 2019; Zheng et al., 2019) to detect putative back-splicing events from fusion reads or split alignment results, whereas other splice-aware aligners (Wang et al., 2010; Zheng and Zhao, 2020) can align circular reads and detect BSJs directly.

Considering that most circRNAs are derived from exonic regions (Ji et al., 2019; Wu et al., 2020) where computational methods cannot accurately distinguish linear and circular reads, the BSJ read count is the most reliable measurement of circRNA expression levels. The BSJ read count is inferred from alignment results, and different filters and statistical strategies have been employed to improve its accuracy and sensitivity (Mangul et al., 2019; Zhang et al., 2020). Alternative approaches using pseudoalignment-based tools for circRNA quantification (Li et al., 2017) can substantially increase the computational efficiency compared with regular alignment-based methods. To compare the expression levels of circRNAs and their host genes, the junction ratio, defined as the ratio of BSJ reads and linear reads mapped to the BSJ site, is often used for comparative analysis. Several computational methods have been developed for accurate estimation of junction ratios (Reimers and Carey, 2006; The Comprehensive R Archive Network, 2022). In addition, circRNAs exhibit alternative splicing patterns, and a number of specific tools have been developed for circular transcript assembly (Gao et al., 2016; Zhang et al., 2016; Wu et al., 2019; Zheng et al., 2019), internal structure visualization (Li et al., 2016; Mose et al., 2016), and differential expression analysis (Zhang et al., 2020; The Comprehensive R Archive Network, 2022). Several comprehensive databases have been constructed for circRNA annotation and prioritization analysis (Dong et al., 2018; Xia et al., 2018; Wu et al., 2020).

## 8 Discussion

As technology advances, RNA-seq methods have become increasingly popular and have revolutionized modern biology and clinical applications, driven by continuous efforts of the bioinformatics community to develop accurate and scalable computational tools. In addition, advancements in sequencing technologies have provided an unprecedented ability to analyze a wide range of biological data, enabling new explorations of novel and existing biological problems. To increase access to RNA-seq methods among new users and young scientists, we provided an overview of the fundamentals of RNA-seq and its associated computational methods and discussed the advantages and limitations of various applications.

Computational analysis of RNA-seq data can be used to tackle important biological problems such as estimating gene expression profiles across various phenotypes and conditions or detecting novel alternative splicing on specific exons. Specialized analyses of RNA-seq data can also help to detect changes in the concentration, function, or localization of transcription factors that affect splicing and can cause the onset of neurodegenerative diseases and cancers (Ozsolak and Milos, 2011; Szabo and Salzman, 2016). Some recently developed computational tools (Xu et al., 2014; Bolotin et al., 2015; Li et al., 2016; Mose et al., 2016; Mandric et al., 2020) are even capable of repurposing RNA-seq data to characterize the individual adaptive immune repertoire and microbiome (Varadhan and Roland, 2008). Additionally, computational deconvolution can be applied to RNA-seq data to study cell-type compositions in tissue samples (Melsted et al., 2017; Kang et al., 2019).

The interdisciplinary nature of RNA-seq applications and related analytic methods and software development introduces a host of terms that can challenge researchers in the wider scientific and medical research communities. The literature on RNA-seq methods has traditionally assumed that readers are familiar with the fundamental concepts of RNA-seq and related bioinformatics analyses (Nariai et al., 2013; Srivastava et al., 2016; Zakeri et al., 2017; Green et al., 2018; Li et al., 2018; Vaquero-Garcia et al., 2018). These methods may require diverse computational skills to be used effectively. A lack of computational skills can therefore limit the ability of biomedical researchers to unlock the full potential of RNA-seq, highlighting the need for a review that explains basic RNA-seq concepts and defines discipline-specific jargon.
