# Supplementary material for: RNA-seq data science: From raw data to effective interpretation
Source: Front Genet. 2023 Mar 13;14:997383. doi: 10.3389/fgene.2023.997383 (PMC10043755; doi:10.3389/fgene.2023.997383)
Supplement: Supplementary file 1 [file DataSheet1.docx]

# Supplementary Materials

**Glossary**

1. Next-generation sequencing: massively parallel sequencing technologies that determine the order of nucleotides in entire genomes or targeted regions of DNA or RNA with ultra-high throughput, scalability, and speed.
2. Third-generation sequencing: technologies that directly sequence a single DNA molecule without amplification, producing substantially longer reads than conventional next-generation sequencing.
3. Complementary DNA (cDNA): DNA produced by reverse-transcription of a template RNA strand (e.g., mRNA) by a reverse-transcriptase enzyme. The cDNA remains single stranded until PCR is carried out.
4. Genome: the collection of all the DNA present in the nucleus and mitochondria of a cell.
5. Transcriptome: the collection of all transcripts, consisting of both non-coding RNA molecules and RNA molecules that encode proteins.
6. Transcript: the single-stranded RNA molecule produced as a result of transcription (copying of a region of DNA into RNA by RNA polymerase).
7. Isoforms: transcripts that are produced from the same gene but have different transcription start sites and potentially different functions as a result.
8. Locus (plural: loci): a specific location or position on a gene.
9. Polymerase chain reaction (PCR): a method used to rapidly amplify or create multiple copies of a specific DNA sample.
10. Nucleotide fluorescence detection: a method of fluorescently labeling each of the four different nucleotide bases with a different color for detection and deduction of the nucleotide sequence.
11. PHRED quality score: a measure of the accuracy of sequencing, or an estimate of the probability of an error in the base call; also called the Q score. This score is provided by the next-generation sequencer.
12. Charge-coupled device (CCD) camera: camera used for digital imaging in sequencing.
13. Poly-A tail: a long chain of adenine nucleotides added to the end of an mRNA for protection against enzymatic degradation.
14. quantitative PCR (qPCR): a way to measure the amplification of reverse-transcribed DNA to determine how much of the original RNA template is contained in a sample.
15. Expression quantitative trait loci (eQTL): genomic loci that account for variation in expression of a specific mRNA or protein.
16. Batch effect: A difference observed among the technical replicates of a sample due to factors such as the day or place where the replicate was sequenced or the researcher conducting the experiment.
17. Normalization: the process of adjusting or removing confounders and variations in data due to batch effects.
18. Single-nucleotide polymorphism (SNP): the replacement of a single nucleotide (base pair) with another nucleotide at a particular position in the genome.
19. Amplification bias: a phenomenon in which there are up to tens of thousands of extra copies of certain reads.
20. Biases of library preparation: a phenomenon that occurs during RNA-seq library preparation when the reverse transcriptase encounters problems such as reading through regions of RNA that have secondary structure or specific base compositions. This is dependent on the reverse-transcriptase primer of choice, which can also bias the results.
21. Noise: variation observed in gene expression data which could be unrelated to the experimental variable of interest and caused due to biological (such as phenotype - age, sex), technical (sample collection, sample processing, sequencing method, technician), or computational (software, parameters) sources of variation.
22. Sequencing errors: sequencing errors are a result of inaccurate base calling by the sequencer (mismatch), or the insertion or deletion of a base (indel).
23. Length biases - the decreased statistical power to detect differential gene expression of shorter mRNAs when compared to long mRNAs.
24. Fragmentation - the process that generates cDNA fragments from RNA transcripts, to produce small pieces (fragments) at specific positions within the transcript, e.g. at the start and/or at the end of the transcript.

# S.1 Supplementary Note 1

**Alternative Splicing:**

The presence of alternative splicing in RNA presents a challenge to aligning RNA-seq reads to a reference genome. The splicing process begins during transcription when DNA is used as a template to create a single strand of RNA. Typically for protein-coding transcripts, the DNA molecule is copied by RNA polymerase into a form known as pre-mRNA, which contains all the complementary bases from the original DNA strand. Next, portions of the pre-mRNA, referred to as introns, are removed, and the remaining portions, referred to as exons, are conjoined to form a mature mRNA. The boundaries between two conjoined exons are called splicing junctions. The final mRNA, referred to as a transcript, is ultimately translated into a protein or serves as a functional non-coding RNA. During the RNA sequencing process, reads are generated from the mature mRNA transcripts, which have already had the introns excised out. To map the reads to the genome, without a pre-compiled list of known transcripts, the reads that span splicing junctions will align to two separate genomic locations that encode the conjoined exons, with the gap between the two exons representing the excised intron.

Alternative splicing is a process where different sets of exons are spliced during the splicing process, leading to diverse mature mRNA transcripts (isoforms) and, consequently, diverse protein products. Alternative splicing is regulated by various splicing factors and introduces additional complexity for analysis, as many isoforms differ in only a few exons. The ratio of isoforms for a gene can be estimated by the ratio of reads that align to isoform-specific junctions **(Figure 2).**

# S.2 Supplementary Note 2

**Sailfish, Salmon, and Kallisto**

Sailfish[^58^](https://www.zotero.org/google-docs/?aVjLzu) pioneered pseudoalignment based on the abundance of k-mers present in each annotated transcript. This reformulation replaces read alignment with k-mer counting, which is considerably faster. K-mers are substrings of length ‘k’ in a nucleotide sequence. For example, a dinucleotide sequence is a k-mer where k = 2. In the sequence CCTAGTGTACCGTACC, CC is a 2-mer with a frequency of three.

Rather than aligning reads and genomic sequences base-by-base, reads can be assigned to genomic regions by matching k-mers between the reads and the genome sequence. For instance, a genomic region with the sequence CCTAGTGTACCGTAC can be represented as a sequence of 5-mers: CCTAG, CTAGT, TAGTG, AGTGT, GTGTA, TGTAC, GTACC, TACCG, ACCGT, CCGTA, and CGTAC. These 5-mers are assigned to the genomic region, and the information is stored in a database for faster lookup. A read with sequence GTACCGT can also be represented as a sequence of 5-mers: GTACC, TACCG, and ACCGT. To determine the genomic origin of the read, the 5-mers in the read are queried from the database. Because all the 5-mers in the read can also be found in the genomic region described above, the read can be assigned to the genomic region without base-by-base alignment.

Most recent methods leverage raw reads to quantify gene expression, are computationally efficient, and use less memory than alignment-based approaches[^53,57,60^](https://www.zotero.org/google-docs/?jMxvJ9). Sailfish vastly accelerates RNA quantification, but it neglects to account for coherence between the k-mers within a read or read pair, which can diminish its accuracy. Kallisto’s algorithm deciphers the compatibility of reads with transcripts[^65^](https://www.zotero.org/google-docs/?2UQUtr) by matching k-mers from reads with a group of transcripts; the intersection of the sets of transcripts represents the best possible matches[^127^](https://www.zotero.org/google-docs/?Ix1RZh). These transcript compatibility sets define equivalence classes of fragments, which are used together with counts as statistics to estimate transcript-level abundances[^128^](https://www.zotero.org/google-docs/?S5NNNw).

Salmon, a method comparable to Kallisto, offers two different modes for quantification of transcripts. One mode includes mapping and quantification in a single step by mapping reads directly to the transcriptome using the quasi-mapping approach of RapMap[^129^](https://www.zotero.org/google-docs/?lXqUPc) without creating any intermediate alignment files[^59^](https://www.zotero.org/google-docs/?aLMkbu). Alternatively, transcriptome-aligned SAM or BAM files, such as those used by RSEM[^54^](https://www.zotero.org/google-docs/?O2UxVY), can be provided. This allows Salmon to build a probabilistic model of the sequencing experiment, estimating the abundance of transcripts.

# S.3 Supplementary Note 3

**Quantification of RNA splicing and sQTL analyses**

Computational tools that capture alternative splicing events in RNA-seq data can use multi-mapped reads to characterize the possible set of transcripts that are missing from the results. Further insight regarding differentially expressed genes can then be obtained by comparing this broader set of transcripts between case and control groups.

Estimation of isoform ratios or exon inclusion levels is currently the standard approach to study splicing events in RNA-seq data. Direct inference based on excised introns is the second most common approach to study splicing events. Splicing events can be quantified using methods such as LeafCutter[^126^](https://www.zotero.org/google-docs/?yHKh2g) or MAJIQ-SPEL[^125^](https://www.zotero.org/google-docs/?kmTbD5). MAJIQ-SPEL is often used together with VOILA (a visualization package) to define, quantify, and visualize local splicing variation in RNA-seq data. LeafCutter[^126^](https://www.zotero.org/google-docs/?dXeo6s) quantifies RNA splicing variation in short-read RNA-seq data by anchoring reads that span an intron to quantify the intron usage across samples. By detecting novel introns, LeafCutter can help researchers avoid the additional burden of isoform abundance estimation[^126^](https://www.zotero.org/google-docs/?flKXBu).

Once the ratios of isoforms, exons, or excised introns are available, splicing levels across different treatment groups or different copy numbers of the minor allele can be compared by differential splicing analyses and splicing QTL (sQTL) analyses. LeafCutter[^126^](https://www.zotero.org/google-docs/?gmSgMv) can detect differential splicing between sample groups and map sQTLs. The ulfasQTL[^130^](https://www.zotero.org/google-docs/?Pp7IDH) and sQTLseekeR[^131^](https://www.zotero.org/google-docs/?UkKc3B) methods are commonly selected to map sQTLs.

**References**:

[53. Trapnell, C. *et al.* Differential gene and transcript expression analysis of RNA-seq experiments with TopHat and Cufflinks. *Nat. Protoc.* **7**, 562–578 (2012).](https://www.zotero.org/google-docs/?KJ42KJ)

[54. Li, B. & Dewey, C. N. RSEM: accurate transcript quantification from RNA-Seq data with or without a reference genome. *BMC Bioinformatics* **12**, 323 (2011).](https://www.zotero.org/google-docs/?KJ42KJ)

[57. Bray, N. L., Pimentel, H., Melsted, P. & Pachter, L. Near-optimal probabilistic RNA-seq quantification. *Nat. Biotechnol.* **34**, 525–527 (2016).](https://www.zotero.org/google-docs/?KJ42KJ)

[58. Patro, R., Mount, S. M. & Kingsford, C. Sailfish enables alignment-free isoform quantification from RNA-seq reads using lightweight algorithms. *Nat. Biotechnol.* **32**, 462–464 (2014).](https://www.zotero.org/google-docs/?KJ42KJ)

[59. Patro, R., Duggal, G., Love, M. I., Irizarry, R. A. & Kingsford, C. Salmon provides fast and bias-aware quantification of transcript expression. *Nat. Methods* **14**, 417–419 (2017).](https://www.zotero.org/google-docs/?KJ42KJ)

[60. Alser, M. *et al.* Technology dictates algorithms: Recent developments in read alignment. Preprint at http://arxiv.org/abs/2003.00110 (2020).](https://www.zotero.org/google-docs/?KJ42KJ)

[65. Li, S. *et al.* Detecting and correcting systematic variation in large-scale RNA sequencing data. *Nat. Biotechnol.* **32**, 888–895 (2014).](https://www.zotero.org/google-docs/?KJ42KJ)

[125. Green, C. J., Gazzara, M. R. & Barash, Y. MAJIQ-SPEL: web-tool to interrogate classical and complex splicing variations from RNA-Seq data. *Bioinformatics* **34**, 300–302 (2018).](https://www.zotero.org/google-docs/?KJ42KJ)

[126. Li, Y. I. *et al.* Annotation-free quantification of RNA splicing using LeafCutter. *Nat. Genet.* **50**, 151–158 (2018).](https://www.zotero.org/google-docs/?KJ42KJ)

[127. van IJzendoorn, D. G. P. *et al.* Machine learning analysis of gene expression data reveals novel diagnostic and prognostic biomarkers and identifies therapeutic targets for soft tissue sarcomas. *PLOS Comput. Biol.* **15**, e1006826 (2019).](https://www.zotero.org/google-docs/?KJ42KJ)

[128. Risso, D., Ngai, J., Speed, T. P. & Dudoit, S. Normalization of RNA-seq data using factor analysis of control genes or samples. *Nat. Biotechnol.* **32**, 896–902 (2014).](https://www.zotero.org/google-docs/?KJ42KJ)

[129. Simoneau, J., Gosselin, R. & Scott, M. S. Factorial study of the RNA-seq computational workflow identifies biases as technical gene signatures. *NAR Genomics Bioinforma.* **2**, lqaa043 (2020).](https://www.zotero.org/google-docs/?KJ42KJ)

[130. Yang, Q., Hu, Y., Li, J. & Zhang, X. ulfasQTL: an ultra-fast method of composite splicing QTL analysis. *BMC Genomics* **18**, 963 (2017).](https://www.zotero.org/google-docs/?KJ42KJ)

[131. Monlong, J., Calvo, M., Ferreira, P. G. & Guigó, R. Identification of genetic variants associated with alternative splicing using sQTLseekeR. *Nat. Commun.* **5**, 4698 (2014).](https://www.zotero.org/google-docs/?KJ42KJ)
